# Prevalence of antibodies against a cyclic peptide mimicking the FG loop of the human papillomavirus type 16 capsid among Tunisian women

**DOI:** 10.1186/s12967-020-02450-5

**Published:** 2020-07-29

**Authors:** Elham Hassen, Devendra Bansal, Randa Ghdira, Anouar Chaieb, Hedi Khairi, Abdelfattah Zakhama, Sami Remadi, Johan Hoebeke, Ali A. Sultan, Lotfi Chouchane

**Affiliations:** 1 Laboratoire d’immuno-oncologie moléculaire, Faculté de Médecine de Monastir, 5019 Monastir, Tunisia; 2 Institut Supérieur de Biotechnologie de Monastir, Université de Monastir, Monastir, Tunisia; 3 Department of Microbiology and Immunology, Weill Cornell Medicine-Qatar, Doha, Qatar; 4 UPR9021 «Immunologie et Chimie Thérapeutiques», Institut de Biologie Moléculaire et Cellulaire, CNRS, Strasbourg, France; 5 Service d’obstétrique et des maladies féminines, Hôpital Universitaire Farhat Hached, Sousse, Tunisia; 6 Laboratoire d’anatomo-pathologie, Faculté de Médecine de Monastir, Monastir, Tunisia; 7 Laboratoire CYTOPATH, Sousse, Tunisia; 8 Department of Genetic Medicine, Weill Cornell Medicine, New York, USA; 9 Genetic Intelligence Laboratory, Weill Cornell Medicine-Qatar, Qatar Foundation, P.O. Box 24144, Doha, Qatar

**Keywords:** HPV16, Antibodies, Cyclic peptide, Major capsid protein L1, Tunisia

## Abstract

**Background:**

In the past decade, cervical cancer has gone from being the second to the fourth most common cancer in women worldwide, but remains the second most common in developing countries. This cancer is most commonly caused by high-risk types of human papillomavirus (HPV), mainly type 16 (HPV16), which are sexually transmitted. This study aimed to investigate the usefulness of a cyclic synthetic peptide designed from the major L1 capsid protein of HPV16 for detecting anti-HPV16 antibodies.

**Methods:**

We designed and synthetized a peptide that corresponds to the full sequence of the surface-exposed FG loop. We tested the antigenicity of the linear and the cyclic peptides against HPV16 L1 monoclonal antibodies. We used ELISA to detect anti-peptide antibodies in sera and cervical secretions of 179 Tunisian women, and we applied polymerase chain reaction and direct sequencing methods to detect and genotype HPV DNA.

**Results:**

Both the linear and the cyclic peptides were recognized by the same neutralizing monoclonal antibodies, but the cyclic peptide was more reactive with human sera. The prevalence of the anti-peptide antibodies in sera was higher in women with low-grade squamous intraepithelial lesions (LGSIL) than in women with high-grade squamous intraepithelial lesions (HGSIL) (44% and 15%, respectively). This contrasts with HPV16 DNA prevalence. Compared to women from the general population, systemic IgG prevalence was significantly higher among sex workers (25%; *P *= 0.002) and women with LGSIL (44%; *P *= 0.001). In addition, systemic IgA and cervical IgG prevalence was higher among sex workers only (*P *= 0.002 and *P *= 0.001, respectively). We did not observe anti-peptide IgG antibodies in women with a current HPV16 infection.

**Conclusion:**

Anti-peptide IgG in sera or in cervical secretions could be markers of an effective natural immunization against HPV16. This may open novel perspectives for monitoring vaccinated women and for the design of synthetic peptide-based vaccines.

## Background

Human papillomavirus (HPV) infection of the uterine cervix is a common sexually transmitted disease. Molecular epidemiological studies have identified high-risk HPV infection as a major risk factor for develop cervical cancer and its precursors [1]. Cervical cancer, after breast cancer, is the second most common cancer in developing countries and remains one of the most frequently diagnosed malignant gynecological cancers worldwide. There were > 570,000 new cases and 311,000 deaths in 2018 [2]. HPV types frequently associated with cervical cancer are classified as high-risk genotypes (16, 18, 31, 33, 35, 39, 45, 51, 52, 56, 58, 59, 66, and 68), while those causing genital warts are designated low-risk HPV genotypes (6 and 11) [3]. HPV16 is the most frequent oncogenic type detected in North Africa and worldwide, while the other types are more frequent in Latin America and Alaska [4, 5]. HPV infection with high-risk types may progress from infection to cervical lesions and cancer [6–9]. The progression of the pathogenesis from low-grade squamous intraepithelial lesions (LGSIL) to high-grade squamous intraepithelial lesions (HGSIL) and cervical cancer is an infrequent event [10], suggesting that the natural course of viral infection may be modulated by the immune response of each woman.

Papillomaviruses are non-enveloped DNA viruses, with an icosahedral capsid of approximately 55 nm diameter. Their double-stranded DNA genomes of 8.0 kb encode Late and Early proteins (L1, L2, E1, E2, E4, E5, E6 and E7) [11–13]. The capsid of a papillomaviruses is composed of the two Late proteins, L1 and L2, the major capsid protein and the minor capsid protein, respectively. The expressed L1 protein self assembles into virus-like particles (VLPs), which are morphologically and antigenically similar to native virions. Several studies have demonstrated that HPV16 VLPs can be used to develop a humoral response against the viral capsid proteins [14–17]. The crystallography model of the HPV16 L1 protein predicts the existence of five loops (BC, DE, EF, FG, and HI) that are displayed on the surface of assembled capsomers [18]. All conformational epitopes identified to date using specific monoclonal antibodies have been found to reside on one or more hyper-variable loops of the L1 protein [19–24].

In this study, we designed and synthesized a peptide corresponding to the FG loop of the major L1 capsid protein that includes residues implicated in the binding of neutralizing antibodies [20, 21]. The benefit of cyclized peptide over linear peptide has been previously suggested, and we applied the cyclization approach to the synthesized peptide because conformation has an important role in peptide antigenicity [25, 26]. We used an Enzyme Linked Immunosorbent Assay (ELISA)-based serological test to investigate the usefulness of the cyclic FG synthetic peptide for detecting HPV16 antibodies.

## Methods

### Study subjects

We included a total of 179 women in this study. These included women with cervical lesion (n = 43), legal sex workers (n = 51), and general population (n = 85) from Tunisia (North-Africa). Forty-three women with cervical lesions were recruited from the department of gynecology of Farhat-Hached Hospital, Sousse. This group includes different clinical stages of HPV lesions before any treatment. According to the Bethesda system for reporting of cervical cytology, women were classified into two lesion groups, LGSIL (n = 16) and HGSIL (n = 27). Fifty-one legal sex workers were included in this study; these women were attending The Primary Care of Sousse on a monthly basis. The study population, design and epidemiological information were described previously in detail [27, 28]. Eighty-five women were randomly chosen from the general population; these women were visiting the department of gynecology of Farhat-Hached Hospital for routine gynecologic examination or to receive contraceptives. The three populations underwent a complete gynecologic examination, including a personal interview, a cervical smear for cytology, a blood sampling, and a cervical sampling. The Ethical Commission of the Ministry of Public Health approved the study and all subjects who agreed to participate in this study gave informed consent.

### Sample collection and cytologic evaluation

The cervical samples were collected with a cytobrush from the transformation zone of the cervix. After obtaining smears for cytology, the remaining cell samples with mucosal secretion on the brush were suspended in 1.5 ml of Hanks buffer sterile solution (HBSS). After vortexing and centrifugation, the pellets were used for HPV DNA testing and the supernatants were stored at − 70 °C for IgG testing. Plasma samples obtained from heparinized blood were also stored at − 70 °C for further use. Before use, plasma was centrifuged at 10,000 rpm for 10 min at 4 °C to remove debris. Smears were screened by experienced cytopathologists who made the final clinical diagnosis of the women with abnormal cytology using histologic evaluation of biopsy samples obtained at colposcopy. Both cytologic and histologic diagnoses were performed according to the Bethesda system.

### HPV DNA detection and sequencing

We extracted DNA using denaturation and precipitation with trimethyl-ammonium bromide salts method. The DNA pellet was dissolved in Tris–EDTA buffer and stored at − 70 °C. HPV DNA detection was carried out, as previously described [27]. Briefly, the presence of HPV DNA was performed using the L1 consensus degenerated primer (MY09/MY11) [28], which targets a conserved 450pb from the L1 ORF (open reading frame) and permits the detection of a large spectrum of genital HPV types. Each 20 μl reaction contained 200 mM of each dNTP, 10 mM Tris hydrochloride (pH 8.3), 50 mM potassium chloride, and 1.5 mM magnesium chloride; 80 pmol of each HPV L1 consensus degenerated primer; 8 pmol of each β-globin primer and 0.5 units of Taq polymerase (Amersham, Paris, France). The cycling conditions for PCR in an automatic thermal cycler (Biometra, Gottingen, Germany) include 30 cycles of denaturation at 94 °C for 30 s, annealing at 55 °C for 60 s, and extension at 72 °C for 90 s. The presence of human DNA was controlled in all samples by PCR amplification specific for the β-globin gene using the PCO3/PCO4 primers [29].

To identify HPV types, HPV-DNA positive samples were subjected to DNA sequencing. We sequenced the gel purified L1-PCR products using BigDyeDeoxy terminator cycle sequencing kit (BD V3.1, Applied Biosystems) according to the manufacturer’s instructions. The products were then purified on a separation column (AutoSeq™ G-50, Amersham Biosciences), and the templates were sequenced on an automated ABI-PRISM 310 Genetic Analyzer (Applied Biosystems). Sequence analysis was performed using nucleotide–nucleotide BLAST analysis (blastn) against known HPV genotypes stored in the GenBank database [30].

### Peptide synthesis and cyclization

The synthesized peptide (L1FG/HPV16) was designed from the FG loop of the HPV16 capsid protein L1. The sequence of the synthetic peptide is given in Table 1. To identify the possible HPV-16 type restricted epitopes in the exposed FG loop, sequences of the most frequently detected HPV types (HPV16, 18, 6, 11, and 31) were aligned. The L1FG/HPV16 was assembled using Fmoc (9-fluorenylmethyloxycarbonyl) chemistry with a multichannel peptide synthesizer [31]. For cyclization of the L1FG/HPV16, a cysteine residue was added to the C-and N-terminus of the selected sequence peptide. After trifluoroacetic acid (TFA) cleavage, the peptides were purified by HPLC and their integrity was assessed by MALDI-TOF spectrometry. Cyclization of the L1FG/HPV16 was done at room temperature overnight by air oxidation of the free thiol groups at 0.1 mg/ml peptide in water, pH 7.5. The cyclized peptide was purified by HPLC and after lyophilization the cyclic peptide was characterized as above.

**Table 1:** Alignment of amino acid sequences corresponding to the HPV16 linear selected peptide

Papillomavirus	Sequences																									Identities (%)
	266																282								290	
HPV16	T	**V**	G	**E**	**N**	V	P	D	D	L	Y	I	K	G	**S**	**G**	**S**	**T**	A	**N**	L	A	S	S	N	100
HPV18	_	M	_	D	T	_	_	Q	S	_	_	_	_	_	T	_	M	P	_	S	P	G	_	C	V	58
HPV31	_	_	_	_	S	_	_	T	_	_	_	_	_	_	_	_	_	_	_	T	_	_	N	_	T	84
HPV6	E	V	_	_	P	_	_	_	T	_	I	_	_	_	_	_	N	R	T	S	V	G	_	_	I	60
HPV11	_	_	_	_	P	_	_	_	_	_	L	V	_	_	G	N	N	R	S	S	V	A	_	_	I	60

Residues in bold designed contact amino acids to H16.V5 [39]; Residues underlined designed overlapping amino acids to H16.V5 and 26D1 [24]

### ELISA reactivity of neutralizing monoclonal antibodies to synthetic peptides

We tested peptide antigenicity using three neutralizing monoclonal antibodies, H16.H5, H16.V5 and H16.E70 [19–21] (kindly provided by Dr. N.D. Christesen). ELISA was prepared as follows:

First, the plates were coated with 500 ng per well of the synthetic peptides in PBS (pH 9.7 for the non-cyclic peptide and pH 7.4 for the cyclized peptide) and incubated overnight at 4 °C. Then, the wells were washed and blocked for 1 h at 37 °C with PBS containing 0.1% Tween-20 and 1% non-fat milk. Next, the plates were washed, followed by the addition of two-by-two dilution of the neutralizing monoclonal antibodies (from dilution 1:2 to 1:128) and incubated for 1 h at 37 °C. After five washes, peroxidase-conjugated anti-mouse IgG diluted 1:2500 was added to the wells and incubated for 1 h at 37 °C. After four washes with PBST and twice with PBS, the antibody binding was revealed by adding 3,3′,5,5′-tetramethylbenzidine (TMB) in the presence of H_2_O_2_.

### Antibody detection by ELISA

Antibodies against the synthetic peptides were quantified by ELISA. Briefly, 96 well microtiter plates (Greiner, Microlon) were coated with 100 ng of the synthetic peptide in PBS (pH 9.7 for the linear peptide and pH 7.4 for the cyclic peptide) per well and incubated at 4 °C overnight. After washing with PBS-0.1% Tween 20 (PBS-T), the wells were saturated with PBS-T supplemented with 1% gelatine (PBST-G) for 1 h at 37 °C. Duplicate wells, one test coated with peptide/PBS and one control with PBS, were incubated with human plasma, diluted 1:50 in PBS-T supplemented with gelatine 0.1% (PBST-G) or undiluted cervical samples for 2 h at 37 °C. The plates were washed five times with PBS-T and bound antibodies were detected with a horseradish peroxidase-conjugated rabbit anti-human IgG or a horseradish peroxidase-conjugated rabbit anti-human IgA (Sigma, St. Louis, MO USA). A monoclonal anti-human secretory component and an anti-mouse IgG horseradish peroxidase-conjugated were used for detection of secretory IgA (sIgA) in the cervical secretion, (Sigma, St. Louis, MO USA). Finally, the wells were washed three times with PBS-T and twice with PBS before revealing antibody binding by addition of ABTS (2,2-Azino-bis-[3-ethylbenzothiazoline-6-sulfonic acid]) at 20 μg/ml in 0.1 M sodium acetate buffer (pH 5) and H_2_0_2_ at 4 mM. Absorbance was read at 405 nm with an automated plate reader (Dynatech Laboratories MRX). The absorbance of each sample on PBS-coated wells (control) was subtracted from that measured on peptide-coated wells (test). To define antibody positive sera, the cutoff value was calculated separately for each peptide as the median of the specific absorbance values of all control sera plus 2.5 standard deviations excluding positive outliers. A corrected absorbance was considered positive at 0.1 or more for serum and 0.05 or more for cervical secretions.

### Statistical analysis

The Chi square and the Fisher test were used to determine significant differences (*P* value). Correlations were assessed using the Spearman rank test. A *P*-value of less than 0.05 was considered significant.

## Results

### Demographic, clinic characteristics and risk factors of studied subjects

All the women enrolled in this study were from Tunisia. The age range across the studied population was 18 to 73 years with a mean age of 37 years. The majority of women from the general population or with cervical lesion (HGSIL and LGSIL) were married (85%) and monogamous. The median age at first intercourse was found to be 19 years (ranges 12 to 27 years). This population was homogenous in terms of social habits. None of the women had history of using contraceptive or condoms systematically, smoking, or drinking alcohol. Out of 43 women with cervical lesions, 16 were diagnosed as LGSIL and 27 as HGSIL. All the sex workers enrolled in the study were legal, relatively homogenous in terms of sexual activity and high exposure risk factors for HPV infection. Fourteen (27.4%) sex workers had experienced first intercourse before the age of 20. The mean of years of sex work was 4 years (ranging 0 to 16 years). The majority of sex workers reported smoking (n = 45, 88.2%) and drinking alcohol (n = 25, 49%). The condom use was reported as follow: 18 women (35%) never, 14 women (27%) sometimes, and 19 (37%) all of the time.

### Binding of the peptides to the N-MAbs

**Fig. 1:**
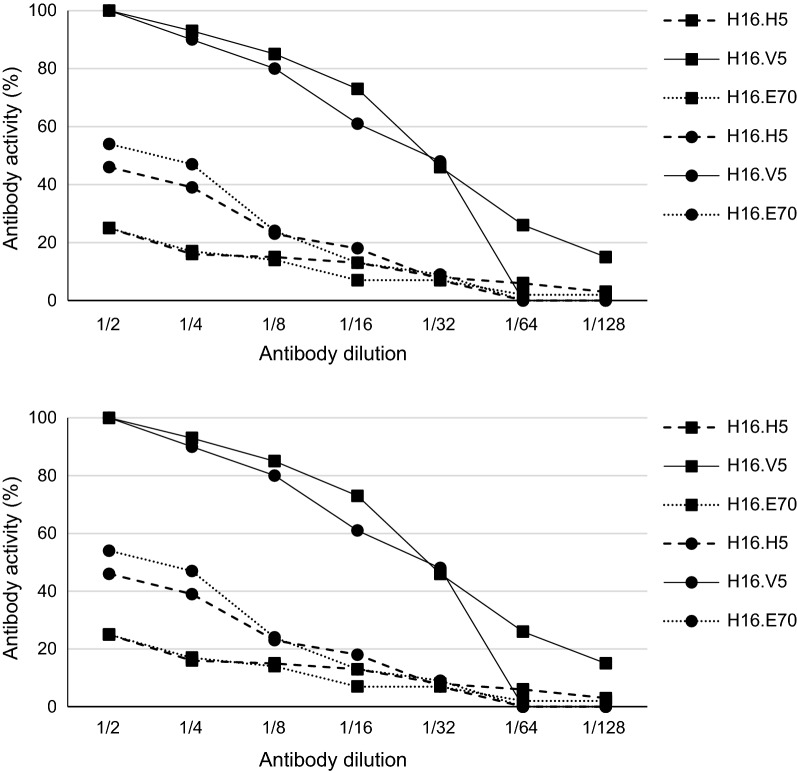
Monoclonal antibody activity against the linear (squares) and the cyclic (circles) of the L1FG/HPV16 peptides

To demonstrate if the linear and the cyclic peptides are recognized by neutralizing antibodies, we tested their antigenicity by using three specific monoclonal antibodies (MAbs) raised against the L1 capsid protein of HPV16 (Fig. 1) [19–21]. The H16.H5 antibody is a murine monoclonal antibody with no neutralizing activity or type specificity. H16.H5 is reactive to a linear epitope including residues from 174 to 185, in the EF loop [19, 20]. The H16.V5 and the H16.E70 antibodies are both neutralizing antibodies that recognize conformational epitopes on the surface of HPV16 VLPs [21]. H16.V5 and H16.E70 recognize different epitopes on the surface of HPV16 VLPs, although there is some overlap in the residues recognized by the two antibodies localized at the FG loop. Both the FG and DE loops were necessary for binding of the H16.E70, whereas the FG loop is the predominant epitope recognized by H16.V5 [21]. Compared to the H16.H5 and H16.E70 antibodies, the H16.V5 antibody was the most reactive against both the linear and the cyclic peptides. Both peptides likely have comparable antigenicity against the H16.V5 antibody. The reactivity curves of the H16.H5 and H16.E70 antibodies were overlapped for each form of the peptides, but lower than H16.V5. However, the cyclic peptide was more reactive than the linear one with H16.H5 and H16.E70 antibodies (Fig. 1).

### Linear and cyclic peptide correlations

**Fig. 2:**
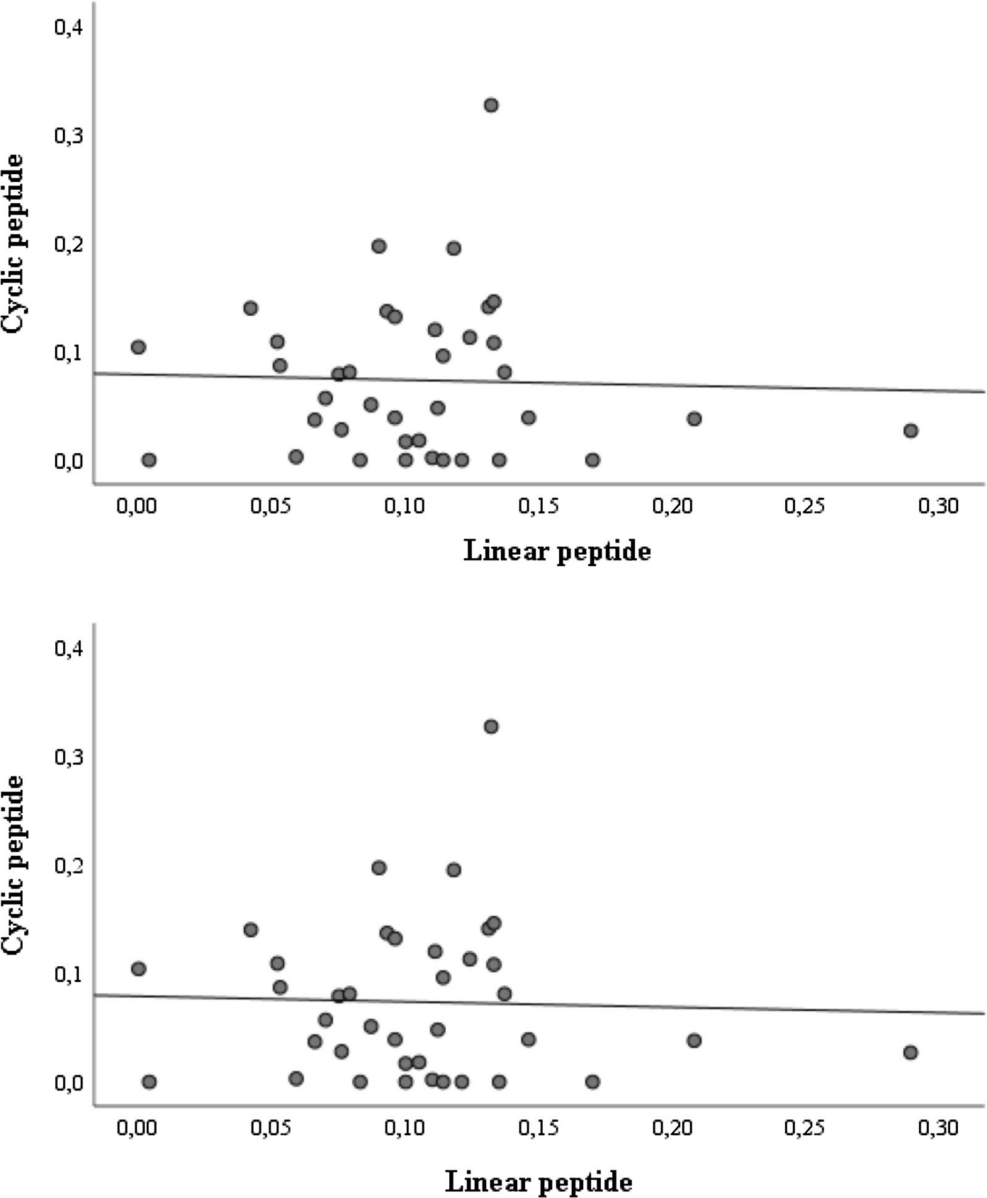
Correlation between linear and cyclic peptide ELISAs for IgG capture from women sera (absorbance at 405 nm). Pearson’s correlation coefficient, r = − 0.036; *P *= 0.830

To show if the two shapes of the peptide (linear and cyclic) have the same antigenicity to human sera, we tested the two peptides against sera samples from several groups of the study population. Pearson correlation analysis of the ELISA optical density results (405 nm) for IgG detection using either linear or cyclic peptides showed no significant correlation (r = − 0.036; *P *= 0.830) (Fig. 2). Unlike what we observed with mouse MAbs the two shapes of the peptides seem to react differently when binding to human polyclonal antibodies. The cyclic peptide is more reactive with human sera. This difference in reactivity may have several reasons. First, antibodies in human sera are directed against a larger panel of epitopes. Secondly, cyclic peptides are known to be more stable than linear peptides with identical amino acid sequences. They are especially resistant to hydrolysis by exopeptidase as they lack amino and carboxyl ends. Besides, the rigidity of cyclic peptides increases binding affinity, which can extend their biological activity and improve recognition of epitope mimics [32, 33]. For all these reasons, only results obtained with the cyclic peptide are presented below.

### Detection of systemic and cervical anti-L1FG/HPV16 antibodies

The distribution of positive women to anti-L1FG/HPV16 antibodies is summarized in Table 2. For statistical analysis, the reference group was the healthy women from the general population. The systemic IgG antibody prevalence was significantly higher among women with LGSIL and sex workers (44% and 25%, respectively); however, comparable frequencies were observed among women with HGSIL and those from the general population (15% and 12%, respectively). The prevalences of systemic IgA and the cervical IgG antibodies were significantly elevated among the sex worker group, suggesting the influence of their lifestyle on antibody production (23% and 16%, respectively).

**Table 2:** Distribution of anti-L1FG/HPV16 IgG and IgA antibodies

	HPV DNA		Systemic IgG		Systemic IgA		Cervical IgG		Cervical sIgA	
	n/total (%)	*P* value	n/total (%)	*P* value	n/total (%)	*P* value	n/total (%)	*P* value	n/total (%)	*P* value
General population*	14/85 (16)		10/85 (12)		5/85 (6)		1/85 (1)		3/85 (3)	
Sex workers	20/51 (39)	*0.005*	13/51 (25)	*0.002*	12/51 (23)	*0.002*	8/51 (16)	*0.001*	4/51 (8)	*0.2*
LGSIL	10/16 (62)	*0.0004*	7/16 (44)	*0.001*	3/16 (19)	*0.1*	2/62 (12)	*0.006*	1/62 (5)	*0.5*
HGSIL	22/27 (81)	<* 0.0001*	4/27 (15)	*0.4*	4/27 (15)	*0.1*	0/27 (0)	*0.7*	0/27 (0)	*0.4*

*LGSIL* low grade squamous intraepithelial lesions, *HGSIL* high grade squamous intraepithelial lesions

* Reference group

The HPV DNA prevalences were significantly higher among sex workers and women with LGSIL or HGSIL (39%, 62%, and 81%, respectively) compared to healthy women from the general population, (Table 2). Moreover, in the overall population study, genotyping results by sequencing showed that the HPV16 was the most frequent type (13%, 23/179), followed by HPV6 (8%, 14/179) and HPV11 (5%, 9/179). The frequency was about 1% (2/179) for HPV18, 53, 56, 58, 66, 68, 84 and about 0.5% (1/179) for HPV31, 33, 45, 61, 70, 81, 82, 83. Co-infection with two HPV types was observed in two cases HPV6/HPV11 and HPV11/HPV18. As antibodies are a marker of past as well as present infection, we examined the relationship between HPV16 capsid FG loop sero-reactivity and the status of HPV16 infection (Table 3). The overall frequency of HPV16 DNA positivity was 13% (23/179). Interestingly, none of the HPV16-positive women showed positive systemic or local IgG anti-peptide antibodies. However, among HPV16 DNA-negative women but infected by HPV types other than the HPV16 (HPV18, 31, 33, 45, 56, 58, 68, 82, 53, 66, 6, 11, 61, 70, 81, 83 and 84) [28], we detected a higher antibody prevalence in both sera and cervical secretions. These results suggest that detection of anti-L1FG/HPV16 IgG antibodies is unrelated to a current infection with HPV16.

**Table 3:** Distribution of anti-L1FG/HPV16 IgG and IgA antibodies according to HPV infection

	HPV	Systemic IgG		Systemic IgA		Cervical IgG		Cervical sIgA	
	n/total (%)	n/total (%)	*P* value	n/total (%)	*P* value	n/total (%)	*P* value	n/total (%)	*p* value
HPV DNA negative*	111/179 (62)	22/111 (20)		12/111 (11)		5/111 (4)		5/111 (4)	
HPV DNA positive HPV16 negative^§^	45/179 (25)	12/45 (27)	*0.3*	8/45 (18)	*0.2*	6/45 (13)	*0.05*	2/45 (4)	*0.6*
HPV16 positive	23/179 (13)	0/23 (0)	*0.01*	4/23 (17)	*0.2*	0/23 (0)	*0.3*	1/23 (4)	*0.7*

* Reference group

^§^HPV positive for the following types: HPV18, 31, 33, 45, 56, 58, 68, 82, 53, 66, 6, 11, 61, 70, 81, 83 and 84 [28]

To identify a prognostic signification of the anti-LlFG/HPV16 antibodies, we extended our analysis and compared results from LGSIL and HGSIL patients. The proportion of local IgG and IgA was very low in cervical samples and could not be compared. However, in sera, the frequency of the antibodies was significantly more elevated among women with LGSIL compared to HGSIL (44% versus 15%; *P *= 0.04). This suggests that women with anti-peptide antibodies have a better prognosis than those without antibodies.

**Fig. 3:**
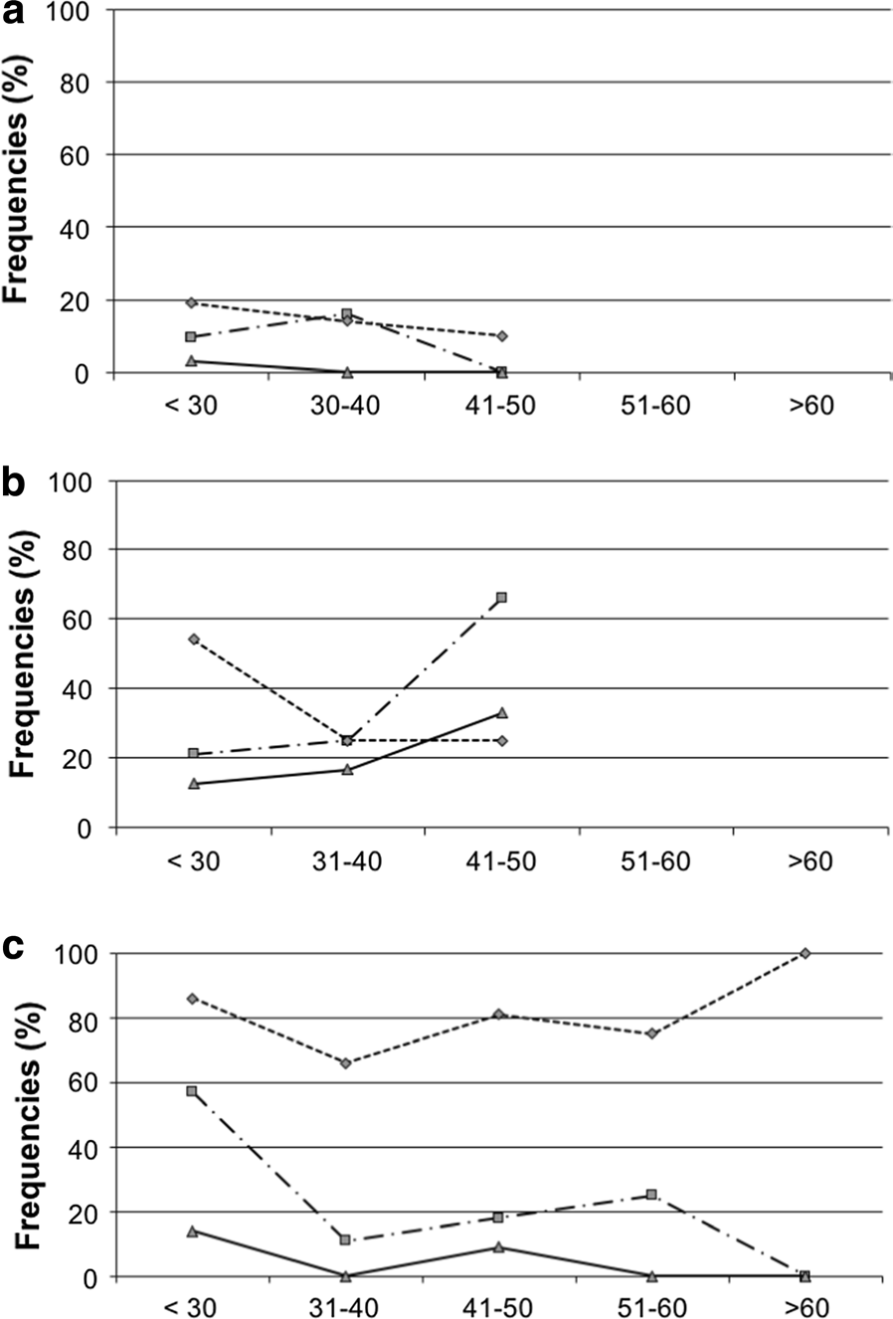
Frequencies of IgG antibodies in sera (squares), in cervical secretions (triangles) and DNA prevalence (diamonds) in all age groups in the women of the study: **a** healthy women; **b** sex workers; **c** women with cervical lesions

We have previously shown that HPV infection decreased with age among healthy women [27, 28]. To assess if we observed the same pattern with the antibody reactivity to L1FG/HPV16, we overlapped the HPV DNA and IgG prevalence curves according to age (Fig. 3). Among healthy women from the general population, the HPV DNA and IgG prevalences were less than 20% and did not change significantly with age. Among the sex workers, the prevalence of systemic IgG increases (21% to 66%), while, conversely, HPV DNA prevalence decreased (54% to 25%) from the age of 31 years. Among the women with cervical lesions, HPV DNA prevalence remained elevated, but the prevalence of systemic IgG markedly decreased from the age of 31 years. Altogether, when we compare the pattern among sex workers and women with cervical lesions, we observed inverted and positive progress, suggesting that anti-L1FG/HPV16 antibodies may have an efficient effect on HPV clearance.

## Discussion

To date, several studies have used synthetic peptides to detect antibodies against the HPV L1 protein [34–37]. However, these studies have used linear synthetic peptides. The FG loop is a hyper-variable region exposed on the outer surface of the virus capsid. Several residues in the FG loop are involved in the binding of neutralizing antibodies [20, 21, 38]. On the basis of these findings, we looked for an amino-acid sequence that could account for a specific epitope into the HPV16 L1 protein. To do this, we aligned the FG loop sequences from the detected HPV types (HPV16, 18, 31, 6 and 11) (Table 1). After determining the percentage of amino acid identity, we selected and synthesized a shorter sequence including 25 residues. It was previously shown that conformation seems to have an important role in peptide antigenicity. The antigenicity of the two forms of the FG peptide were shown by binding to the neutralizing monoclonal antibody H16.V5. The H16.V5 is a HPV16 murine monoclonal antibody, and its neutralizing activity was described before during the publication of the crystallography model of the HPV16 L1 protein [21]. Through a cryo-electron microscopy study on VLP capsid, 17 residues were found to bound to the H16.V5 MAb: eight out the 17 bound to the FG loop (Table 1), two were bound to the BC and DE loops of the same L1 protein, and three and two were bound the DE and HI loops, respectively of the adjacent L1 protein [39]. However, the characterization of a human neutralizing antibody 26D1 showed some neutralizing epitopes that were distinct from those of H16.V5. The 26D1 human monoclonal antibody recognizes a conformational epitope that partially overlaps that of H16.V5. Regarding the FG loop, out of the eight binding residues of the H16.V5, only three residues were overlapping with the 26D1, suggesting that human neutralizing MAbs could have different interaction with the VLP capsid [24]. To obtain the best mimic of the FG loop, we added two cysteine residues at the N- and C-terminus of the peptide, and we applied a cyclization step. As suggested by molecular modeling, the cyclization of the FG linear peptide might induce the native conformation of the FG loop. Moreover, cyclic peptides are more resistant to hydrolysis by peptidase and more rigid to ensure better affinity and recognition of epitope mimics [32, 33]. When we tested the antigenicity of the linear and the cyclic peptides with human sera and compared the reactivity of the linear peptide and the cyclic peptide, the results did not match between the two peptides. The difference in stability and rigidity between the two peptides could be the cause that confirms the importance of cyclization.

We used ELISA to quantify the antibodies raised against the cyclic FG loop in women sera and cervical secretions. The highest seroprevalence (44%) for the systemic IgG was found in women with LGSIL. However, women with HGSIL showed a seroprevalence of 15%, which is comparable to that observed among healthy women (12%). In an earlier study using a linear peptide from residues 267 to 286, weak reactivity was observed in the sera of HPV16 infected women with cervical lesions [34]. In addition, antibodies against L1 peptide from residues 279 to 293 were detected in sera of patients with cervical lesions but did not correlate with HPV16 DNA [35]. The low seroprevalence against the peptide in women with high-grade lesions might be due to an decrease in the L1 expression. Indeed, it is known that L1 protein expression in vivo is usually restricted to low-grade lesions [40]. The transcription of the L1 gene is restricted to differentiated keratinocytes, and the level of L1 expression decreases with the grade of the cervical lesions [41]. These findings indicate that the seroprevalence to these L1 epitopes is unlikely to have a prognostic value.

Interestingly, whatever the women group, in all the HPV16 DNA-positive carriers we did not observe positive IgG in sera or cervical secretion samples. However, women without HPV infection or infected with HPV types other than HPV16 showed prevalence of 22% and 7% in sera and cervical secretion, respectively. A most likely explanation is that seropositive women have been infected previously by HPV16 and that the antibodies detected were those that persist after the virus has been eliminated by the immune system. In contrast, seronegative and HPV16 DNA-positive women were likely unable to produce antibodies against the FG loop. These findings suggest that the anti-L1FG/HPV16 antibodies could have efficient effect on HPV16 infection. Anti-L1FG/HPV16 IgG antibodies in sera or in cervical secretions could be markers of an effective natural immunization against HPV16. Previous observations clearly established that the FG loop contains neutralizing epitopes [42, 43] and a cell-binding region used by HPV for invading target cells [44]. These combined with our results could incite for more investigation on peptide helpfulness in HPV-related infection.

Secretory IgA provides the first line of defense against pathogens invading mucosae, so the levels of these antibodies may provide a better indicator of HPV infection. Several reports that use HPV16 VLPs as antigen suggest that the cervical IgA response reflects current HPV infection [45–48]. However, in this study, we found that positivity was not significantly different and that among the 23 HPV16 DNA positive women, only one was positive for the secretory IgA. This dissimilarity may be due to the fact that anti-VLP antibodies were directed against a multitude of conformational epitopes including those directed against the L1FG/HPV16, meaning that an anti-peptide HPV-negative woman could be anti–VLP positive [49]. An alternative explanation is that in previous reports [45–48] total IgA was detected in the cervical secretions, whereas we quantified only secretory IgA.

We previously demonstrated that the prevalence of HPV infection is strongly associated with risk factors such as sexual activity and age [27, 28]. To find out whether the presence of antibodies was also influenced by these risk factors, we compared the antibody response of women with lesions, sex workers, and women from the general population. The positivity of serologic IgA and cervical IgG was found to be significantly higher in sex workers than in women with lesions or from the general population. This suggests that lifestyle could impact HPV antibodies production. Besides, the IgG response tended to decrease with age in women with cervical lesions and increase with age in legal sex workers, whereas the prevalence of HPV DNA detection remained the same in women with cervical lesions and decreased in legal sex workers (Fig. 3). In a previous study, we found that the decrease in HPV DNA positivity with increasing age was independent of sexual activity but could be related to the development of an efficient immune response acquired with age [27, 28]. Here, this theory is corroborated by the increase of anti-HPV16 IgG responses with increasing age noticed in the sex workers, as distinct from the women with cervical lesions. Sex workers have a high number of sexual partners and repeated risk of HPV infection that may stimulate an anti-HPV immune response that leads to the production of antibodies.

To sum up, by using the L1FG/HPV16 peptide as a capture antigen, we noted: (i) low IgG seroprevalence among women with HGSIL; (ii) nul IgG seroprevalence among women carrying the HPV16 DNA; and (iii) an increasing IgG seroprevalence, accompanied by a decrease in HPV16 DNA prevalence, in women at high risk of exposition to HPV. Taken together, these observations comfort the hypothesis that anti-peptide antibodies could be a marker of good prognostic for the disease and protection from infection. However, for a better estimation of the usefulness of the L1FG/HPV16, we need to conduct the study on vaccinated women and women with cervical carcinoma.

## Conclusion

To the best of our knowledge, this is the first report using a cyclic peptide of the full-length FG loop sequence of the HPV16 L1 protein. The HPV16 is the most prevalent oncogenic HPV type in high grade cervical lesions. While the studied population size needs to be increased, we found that all the women who had developed a high-grade cervical lesion caused by HPV16 failed to produce anti-L1FG/HPV16 antibodies. These findings could have obvious implications for the development of an L1 polycyclic peptide or a new molecular scaffold for peptides that could be an attractive perspective for ELISA testing and a cyclic peptide-based vaccine [33, 50].

## Data Availability

The datasets used and/or analyzed during the current study and patient information sheet are available from the corresponding author on reasonable request.

## Abbreviations

HPV: Human papilloma virus
HGSIL: High-grade squamous intraepithelial lesion
LGSIL: Low-grade squamous intraepithelial lesion
ELISA: Enzyme linked immunosorbent assay
HBSS: Hanks buffer sterile solution
ORF: Open reading frame
